# Men’s perception of barriers to women’s use and access of skilled pregnancy care in rural Nigeria: a qualitative study

**DOI:** 10.1186/s12978-019-0752-3

**Published:** 2019-06-21

**Authors:** Sanni Yaya, Friday Okonofua, Lorretta Ntoimo, Ogochukwu Udenigwe, Ghose Bishwajit

**Affiliations:** 1 School of International Development and Global Studies, University of Ottawa, Ottawa, Canada; 2 Women’s Health and Action Research Centre, Benin City, Nigeria; 3 University of Medical Sciences, Ondo City, Ondo State Nigeria; 4 Centre of Excellence in Reproductive Health Innovation (CERHI), University of Benin, Benin City, Nigeria; 5 Federal University Oye-Ekiti, Oye Ekiti, Ekiti State Nigeria

**Keywords:** Male perception, Barriers to healthcare access and use, Maternal health, Maternal morbidity, Role of men, Global Health, Nigeria

## Abstract

**Background:**

Greater paternal engagement is positively associated with improved access to and utilization of maternal services. Despite evidence that male involvement increased uptake of maternal and child services, studies show that few men are participating in MNCH programs. Community leaders have long been engaged in public health promotion in rural settings and have been shown to mobilize communities to enhance changes in cultural practices related to public health. With the ultimate goal of increasing men’s involvement in maternal health, this study seeks to understand men’s perceptions of community and health systems barriers to maternal access and usage of skilled care in rural Edo, Nigeria.

**Methods:**

This qualitative study involved the analysis of data collected from community conversations with male elders in Etsako East and Esan South East Local Government Areas of Edo State, Nigeria. Community conversations participants (*n* = 128) comprised of elders between the ages of 50–101. A total of 9 community conversations were conducted. Discussions were audio recorded, transcribed and imported into Atlas.ti 6.2 for content analysis.

**Results:**

Men’s perceptions of barriers to maternal use of skilled care are presented in two overarching themes: community systems and health systems. Three sub themes were generated as community systems barriers to maternal healthcare use, they include: gender roles, traditional treatment and policy changes. Three sub themes emerged under health system barriers and they include: cost of health facilities, dissatisfaction with facilities and distance from facilities.

**Conclusion:**

Findings suggest that community elders are not only in a good position to influence men’s behavior, they are also a source of information to policy makers on strategies to overcome barriers to maternal health, especially at the community level. Furthermore, community elders need support to enact regulations that will promote men’s involvement in maternal health, thereby increasing maternal use of skilled care.

## Plain English summary

Current estimates of maternal mortality in Nigeria are some of the highest in the world. The country was listed as one of six countries accounting for 50% of global maternal deaths. Research shows that active involvement of men during pregnancy, child birth and post-partum support improved self-care of the woman and is positively associated with improved access to and utilization of maternal services. Despite evidence that male involvement increased uptake of maternal and child services, studies show that few men are participating in maternal, newborn, and child health programs. Community leaders have long been engaged in public health promotion in rural settings and have been shown to enhance changes in cultural practices related to public health. This study seeks to understand men’s perceptions of community and health systems barriers to maternal access and usage of skilled care in rural Edo, Nigeria and fills a gap by providing rich descriptions of men’s perceived barriers to maternal healthcare access and utilization. Our results suggest that community elders are not only in a good position to influence men’s behavior; they are also a source of information to policy makers on strategies to overcome barriers to maternal health, especially at the community level. Furthermore, community elders need support to enact regulations that will promote men’s involvement in maternal health, thereby increasing maternal use of MNCH services.

### Background

Maternal mortality at the global scale remains unacceptably high with roughly 303,000 women dying each year from pregnancy related complications [1]. Approximately 99% of these deaths occur in low resource settings. Strategies for reducing maternal mortality, especially for the poorest of populations have focused on health systems strengthening. In 1978, the Alma-Ata Declaration identified primary healthcare (PHC) as a key to the attainment of the highest level of health because it is the first level of contact for individuals, families and communities within a country’s health system [2, 3]. Decades later, there remains a continued emphasis on interventions that can be delivered in community settings and at first-level facilities. In 2005, the United Nations Task Force on Child and Maternal Health positioned health systems at the centre of addressing maternal mortality. The Task Force prioritized the strengthening of the primary healthcare system, from first-referral level facilities to the community level [4].

Since 2007, Nigeria’s maternal, newborn, and child health (MNCH) policies have been guided by the Integrated Maternal, Newborn, and Child Health (IMNCH) strategy which echoes the UN taskforce’s priority in its objective to increase maternal access to and utilization of primary health care services [5]. This policy focused on providing services to marginalised women through pregnancy, childbirth and the postnatal period. Within the country’s three-tiered health care system, the Nigerian Ministry of Health implemented the IMNCH strategy through its primary health care system. With a continued focus on the PHC system, two different but complimentary programs; Midwives Service Scheme (MSS) and SURE-P Maternal and Child Health Project [6, 7], were introduced in 2010 and 2012 respectively. MSS tackled the supply side of access to and utilization of PHCs and SURE-P focused on the demand side. Their strategies to improve access and utilization included; increasing human resources, upgrading facilities’ infrastructure, providing and retaining adequately trained midwifes, and cash incentives for use of services. Furthermore, these programs encouraged participation by targeting individuals, families and communities as liaisons between primary health care centres and pregnant women. Evidence from recent family and community level interventions indicate that maternal and neonatal illnesses and mortality can be reduced through such approaches [2].

Community engagement is critical in improving access to health services because various factors, such as, culture and religion affect decision making and health seeking behaviours of individuals in a community [8]. Community leaders, such as elders, are considered positive role models and can help facilitate interventions, particularly those that aim to influence behavioural changes in men and women. They have been shown to be instrumental in promoting positive behaviours among men [9]. The evolving role of men in families continues to be of growing interest as broadened parental roles confront men’s position as solely economic providers and protectors. The growing recognition of men’s critical role in women’s reproductive health calls for more male involvement in maternal health [10, 11]. Alio et al. defines male involvement as a father’s accessibility, engagement and demonstration of responsibility and support (financial, physical and emotional) towards the pregnant mother and unborn child [12]. Numerous studies conducted in the Global North and Global South have demonstrated that male involvement in maternal and child health offers benefits [13]. Greater paternal engagement was positively associated with improved maternal access to and utilization of antenatal services; male involvement during pregnancy improved maternal mental health and decreased the likelihood of risky behaviour (such as smoking); and inter-spousal communication about contraception increased adherence to contraceptive use [14–19]. Similarly, the lack of male involvement can negatively impact women’s access and use of health services. For example, HIV test refusals were high among pregnant women whose male partners did not accompany them to HIV/AIDs prevention programs [20, 21].

### Nigerian context

Nigeria is the second largest contributor to maternal mortality worldwide and has a birth rate of five children per woman [22]. The country’s estimated 58,000 maternal deaths per year accounts for 19% of the global total pregnancy-related deaths [1, 23, 24]. Furthermore, maternal deaths are twice as likely among women in rural areas, compared to women in urban areas. This study is based in Nigeria where patriarchal societies are common. Men are often the heads of households and act as gatekeepers [25–27]. They tend to have exclusive control of their household’s economic resources and are decision makers in all aspects of women’s reproductive health. Men largely determine women’s access to modern health facilities and the availability of resources for maternal health related expenditure. Pregnancy and childbirth have long been considered a woman’s responsibility, therefore, male involvement in the Nigerian context, is a relatively new approach to health integration. So new in fact that the country’s IMNCH strategy, launched in 2007, initially focused on women and does not actively acknowledge the role of men as decision makers on women’s access to health resources [6, 28]. With increased recognition of men as driving forces in women’s access to and utilization of maternal health services, more recent programs such as the Maternal And Child Survival Program (MCSP) are integrating gender into mainstream MNCH services and calling for male involvement in maternal and child health initiatives [29].

Despite evidence that male involvement increased uptake of maternal and child services, studies show that few men are participating in MNCH programs [30, 31]. Reasons for low rates of male involvement have been attributed to societal and health system barriers. These barriers include gender stereotypes, ambiguous roles for men in maternal and child health, poor health facilities and environment and women’s disapproval of men’s involvement. Relational gender theory explains gender as a simultaneous occurrence of economic relations, power relations, affective relations and symbolic relations [32, 33]. These relations give rise to social structures which determine everyday social practices. Gender is therefore enacted within these structures where socially constructed roles and behaviours are ascribed to men and women. Gender-aware interventions recognise that health behaviours are embedded in social structures [34]. Strict gender-based division of labour limits the ways men can engage with pregnancy care, childbirth and even child rearing. Finding an effective way to involve men in pregnancy and childbirth requires an understanding of the influence of their community on the social construction of gender roles. Studies have shown that overcoming those barriers can be achieved by involving community leaders. In rural communities, community leaders and traditional structures remain influential [35]. Community leaders have long been engaged in public health promotion in rural settings and have been shown to mobilize communities to enhance changes in cultural practices related to public health. For example, in Ghana and Malawi, community leaders have been credited with increasing male involvement in pregnancy and maternal care which in turn increased facility-based deliveries [36, 37].

Improved health is a result of a wide range of actors including but not limited to, public health systems (facilities and health care professionals) and community systems (community groups, leaders and community norms) [38]. While not often clearly delineated as a “system”, entities that make up community systems are closer to the community and can better identify, understand and respond to health issues that a community is plagued with. Community systems are therefore crucial in ensuring that programs delivered to community members are comprehensive and meet the needs of the community. In interweaving both community and health systems, marginalised and vulnerable individuals in the population are supported to gain access to their health services to meet their needs.

The preceding information provides an opportunity to reflect on men as part of the broader community system. Men’s critical role in women’s reproductive health has been acknowledged. They are active actors in the community whose roles and involvement in MNCH influence maternal usage of skilled care. In Nigeria, various studies have explored male involvement in maternal care [39–41]. There remains a dearth of studies on men’s perception of barriers to maternal access to and utilization of MNCH services in rural Nigeria. This study therefore seeks to understand men’s perceptions of community and health systems barriers to maternal access and usage of skilled care in rural Edo, Nigeria.

## Methods

### Study design

This study was conducted using an interpretive description design. As a qualitative methodology, interpretive description is located within existing health-related knowledge of researchers and participants, which serves as a foundation for new inquiry [42]. It rejects the notion of a single rigid reality and assumes instead, context bound and inter subjectively constructed realities through social interactions [43, 44]. Interpretive description provides patterns and variations for individual and common human experiences through which researchers and participants co-construct a narrative that can inform clinical practice. This paper draws on qualitative data collected in rural areas of Edo State, Nigeria with a focus on understanding the community and health actors that serve as barriers to maternal access and use of MNCH services. This is a part of a larger original study conducted in rural Edo state, which seeks to identify barriers to equity of access, accessibility and utilization of primary health care services (PHCs) for maternal and newborn health care. The larger study is designed as a community based, multi-site cluster randomized trial using a mixed method approach; a randomized control trial (RCT) and analysis of qualitative data from focus groups, key informant interviews and community conversations (CC) with male elders.

### Setting

Nigeria is Africa’s most populous country and has a population of 180 million people. With an annual population growth rate of 3%, Nigeria is projected to have the second-largest population increase in the world by 2050 [45]. The country is divided into six geopolitical zones and made up of 36 States. About 50% of Nigeria’s population reside in rural areas [46]. This study was conducted in Edo State, one of Nigeria’s thirty-six States. More specifically, the study was carried out in Esan South East and Etsako East local government areas (LGAs) of Edo, both of which are in the rural parts of the State.

### Study participants

Male elders from the community were targeted participants in this study and the reason is two-fold. First, the authors acknowledge that achieving positive social change within a community requires identifying and involving institutions within which powers are exercised [47]. In rural communities such as Esan and Etsokan, the sphere of influence of community elders remains strong. They are the gateway to the community and ensuring their buy-in improves the trust relationship between the communities and researchers. Second, male elders can be instrumental in reconstructing cultural norms within which men’s attitudes towards maternal health are formed. There is evidence that community leaders have helped overcome the reluctance of adult males to participate in maternal health related programs. Therefore, while it is easier to only engage men or husbands of pregnant women, it is important to engage men in communities who have the power to influence community attitudes [48]. Furthermore, this study acknowledges that male elders have the influence to provide a supportive social environment wherein men are encouraged and not stereotyped, when they deviate from traditional male behaviour.

Participants were recruited through purposeful sampling, which considered locally accepted ways of communication. The primary inclusion criteria were age 50 and over and recognition in the community as an opinion leader. Recruitment was continued until data saturation was reached [49]. In rural communities such as Esan and Etsako, face-to-face communication is not only a common means of transferring information, but also a means for governance and social interactions. A gatekeeper who is an indigene of the communities helped to identify elders who meet the inclusion criteria and invited them to the conversation through face-to-face contact. The consent of the traditional ruler in each community was sought and obtained before each CC was conducted.

### Data collection and procedures

This study conducted community conversations with male elders in the community. This methodology is participatory, facilitates buy-in on issues deemed culturally sensitive, and is potentially transformative; preparing supportive groundwork for a useful intervention that would raise awareness and mobilize social change. Studies have shown that community conversations can be agents of change. This approach has helped raise awareness and create consensus about other culturally sensitive issues including HIV testing and prevention, human trafficking, female gender mutilation, and child marriage [50]. We believe that the open and public dialogue could challenge and shift community-level norms about gender and maternal health. The conversations were designed to explore community elders’ understanding of maternal health, community and systems barriers to access and usage of skilled pregnancy care and to proffer potential solutions. Community conversations encourage reflection and discussion; it assumes a conversational format to explore the interpretation barriers to maternal health and ways to reduce it.

The CCs carried out by trained investigators were conducted in Pidgin English and a few in the local language. The fieldwork took place from July 29 to August 162,017. A total of 9 CCs were conducted with 6 in Esan South East LGA and 3 in Etsako East LGA. The number of participants in each CC ranged from 12 to 21. Discussions in the CCs lasted between 60 and 90 min and ended when no further issues arose (point of saturation).

Conversations were audio-recorded, transcribed verbatim and reviewed for clarity and accuracy of the transcription. Any identifying information for each participant was altered to protect their privacy; they were assigned unique codes instead.

Participants’ responses were either transcribed verbatim if they responded in English or translated if they responded in Pidgin English and the local language. Translated quotations are noted in square braces. Literal translation (word-by-word) was used to preserve participants’ responses and provide readers with an understanding of the mentality of the participants [51]. To improve trustworthiness of the data, the data was transcribed by co-authors who are proficient in both Pidgin English and English language. Other coauthors with proficiencies in both languages re-examined the translated transcripts and screened it for errors.

### Issues discussed

A guide was prepared for the CCs which focused specifically on men’s perception of barriers to women’s use and access of skilled care. A sample of some of the issues discussed with participants during the CCs includes:

- Maternal deaths and morbidity in the community and under-5 health
- Perceptions about barriers to use of skilled care during pregnancy
- Perceptions about solutions

### Ethical considerations

The ethical clearance approval needed for the project was obtained from the National Health Research Ethics Committee (NHREC) after the submission of the study protocol. The project ethical clearance certificate was approved on April 18, 2017, with NHREC Approval Number: NHREC/01/01/2007–18/04/2017. To ensure confidentiality, all personal identifiers were removed from transcripts. Written informed consent was obtained from all participants prior to their participation.

### Data analysis

In keeping with the interpretive design of the study, data analysis was iterative and on going throughout the data collection phase, with each process informing the other. Thematic exploration of participants’ perceptions was carried out through a holistic and line-by-line reading of the transcripts. The authors immersed themselves in the transcripts and recordings to identify themes. Given the iterative nature of the data analysis, saturation was achieved when no more patterns or themes emerged from the data. The authors endeavoured to capture and present a holistic understanding of community and health system factors that hindered maternal healthcare access and use.

An open coding was done with Atlas.ti. A code list was first generated deductively from the literature and additional codes were added to the list from the themes that emerged from the data. The codes were merged to generate themes which were classified into two broad categories for the narrative: 1) community systems barriers with themes such as gender roles, and traditional treatment among others, and 2) health systems barriers with themes such as cost of service, dissatisfaction with health facilities etc.

## Results

### Characteristics of study participants

A total of 128 men aged 50–101 years participated in the conversations. Most of them attained post-primary education (63%), whereas a few had no education. The majority were farmers and artisans (56%). Most of the participants were Christians, and a few declared no religious affiliation.

### Community systems barriers

Men’s perception of barriers to maternal healthcare use is presented in two overarching themes: community systems and health systems. Three sub themes were generated as community system barriers to maternal healthcare use, they include: gender roles, traditional treatment and policy changes. Three sub themes emerged under health system barriers and they include: cost of health facilities, dissatisfaction with facilities and distance from facilities.

### Gender roles

Men’s perceptions of barriers to maternal use of MNCH services were identified at the household level. Knowledge pertaining to maternal health was generally considered to be women’s responsibility. Therefore, adequate knowledge on family planning and contraceptive use was lacking among male participants. Participants suggested that women’s ignorance on health issues hindered their use of maternal health services.

*The issue of family planning is for women and most women here have not heard of it. What I think it can be done is to create awareness for them to have knowledge of it, it is their responsibility for them to know when and where to take it and should be brought to their notice because most women don’t know what family planning is so that there will be erratic nature of the knowledge because you don’t give what you don’t have [CC07, Esan South East].*

*So that’s prevention. Fine. That’s all about family planning I know [CC04 Esan South East].*

*Most of our wives don’t know about family planning…they give birth until they are tired of giving birth that is how most of them do family planning [CC07, Esan South East].*

Women’s use of MNCH services was dependent on their husbands’ ability to afford it. While the lack of financial resources was identified as a barrier to accessing and utilization MNCH services, it was always framed in reference to men’s and not women’s financial status.

*At least, the women will adhere to it even some of us don’t have money. We see some pregnant women whose husband cannot afford to register their wives while pregnant. So, going to the traditional care center becomes an option [CC03, Etsako East]*

Some men indicated having no interest in getting involved in certain aspects of maternal health, particularly childbirth. However, men displayed a general understanding of actions that could endanger women’s health during pregnancy.

*You see negligence of some women, too much labour sometimes contributes, you see if a woman is pregnant, at the early trimester, the woman is not supposed to bend down to press the stomach, all these contribute to the over burdening of the woman, in a nutshell* [CC05, Esan South East]

### Traditional treatment

Participants opined that some women preferred traditional treatment. They called for more education to improve awareness on the benefits of using skilled health facilities instead.

*You mentioned one thing then, that you will assemble women and talk to them on the importance of all these things. Awareness still matters. For example, some women here now, when they want to give birth they will be looking for native treatment…but when it results to transfer of blood, sometimes the woman could pass on. So, we will endeavor to gather our women so that you can talk to them and give them awareness with respect to that side [CC05, Etsako East].*

Participants believed that women resorted to traditional care for pregnancy and delivery due to poorly equipped healthcare facilities in their communities.

*These women it is not really that they like going to native homes, it is because there are no facilities in the health center that is why many of them deliver in the native homes. So that is all for now. Thank you [CC05, Esan South East].*

Another participant stated.

*Because some of them instead of going to the hospital they will go to private native hospital like we have here to take treatment. Those people don’t know anything, but they claim they know much. The reason they [women] don’t use the health center is because they don’t have facilities, so they prefer using the native, and there it is trial by error, the woman will struggle continuously and give birth [CC04 Esan South East].*

Some participants believed that both traditional and evidence-based methods of healthcare should be used in tandem. They expressed greater confidence in women’s health status when the two methods of care were sought during pregnancy.

*For me, when I got married and my wife was pregnant, I registered her in general hospital, and also in a traditional Centre. Because my understanding is that, there are medications in the hospital and also another type of medications from the traditional. Because when is time for her to get the traditional medications, she will get it, and when is time for her to get the once from the hospital, she will get them too and if she has any complain of whatsoever, she will tell them and they will give her medications [CC01, Esan South East].*

### Policy changes

There was a general support for MNCH among the participants. Participants repeatedly called for education on MNCH among their communities. They generally perceived that women were unaware of the necessary precautions during pregnancy.

*So, doctor, you said how to get the solution is what brought you here. The solution is education. Most of our women are not educated even if you tell them to go to the hospital, they won’t go. They will only go at the dying minutes. Sometimes there are complications. They need to be enlightened to go for antenatal to monitor them, so that the baby and pregnant woman can be cared for in case there’s any upcoming complication. So, hence you came for the solutions, we have men and women here now, [it] is to enlighten them to go to the hospital for antenatal during pregnancy period. If we can take this step, this issue of death during childbirth will be abolished my major contribution here is educating the woman which is one most important tool [CC01, ESE].*

A participant reported on the improper use of mosquito nets, which were intended to prevent malaria, particularly among women and children. He suggested imposing fines when people defaulted on preventive measures during pregnancy.

*Then another thing, you talked about women’s death during pregnancy and one of the things responsible for this is malaria, because it’s much more difficult to treat a pregnant woman of malaria than it is to treat me, because there are some drugs when she has already taken in can no longer work… if I tell you what our people use net for, it will marvel you. Some of them use them to plant tomatoes, we use them to safe guard tomatoes, we just put them, for where we transplant them, we use them to safe guard them, I know in some communities it is now an offence to use a net to do that, you pay as much as ten thousand naira if you are caught, so also need to educate our people on that [CC01, Etsako East].*

### Healthy systems barriers

The participants identified health systems barriers that not only hindered maternal use of facilities but also hindered men’s involvement in MNCH.

### Cost of service

Participants cited the high cost of health facilities as a deterrent to maternal access and use of facilities. One participant felt that the cost of delivery should depend on the presence or absence of complications during delivery.

*Yes, the charges are too high. Just last month here my daughter paid 8,000 for her last baby…and nothing was wrong with her. They just collect 8,000 like that. She is not supposed to pay 8,000 since she delivered normal. I don’t know why she paid that amount to me is expensive [CC02, Etsako East]*.

The cost of delivery was stated explicitly as a reason for not patronising skilled health facilities.

*Yes, the charges is too high because here. When a woman give birth to a male child they charge 10,000 and when is female is 8,000 so is high that is why we decide not to go again we don’t have that amount to be spending since you people want to come to our aid we are so happy [CC08, Esan South East].*

### Dissatisfaction with health facilities

An overwhelming majority of participants expressed displeasure at the state of healthcare facilities. Issues identified include habitual absenteeism among nurses, poorly equipped health care facilities and unskilled nurses. There were reports of constant absenteeism among nurses, which resulted in low utilization of health facilities.

*The habit of absenteeism is very common among them. Let’s say you ask a nurse to wait for you she will go to the market till late hour before she comes back or the next day [CC07, Esan South East].*

*We know the problem of this health center you have seen it, we all know it. Manpower is number one…secondly; I am a witness to that, even my own wife delivered there, before my wife delivered my second son! It was Suru hospital they rushed her to, because the real midwife who was supposed to be there was not around, manpower, many of them posted down here, they don’t come to work and the drugs are not enough [CC05, Esan South East].*

Participants felt that habitual absenteeism among nurses was more of a problem than the cost of using the facility. Some of the men were willing to pay for quality service if it was available.

*The reason is that any woman that goes to the health centre seat on a chair for hours at the end. No care. Will she not go to where she will be cared for proper[ly]? That is why people don’t patronise them. Is not because of the charges I have never see anyone who come back with good attention and complain that the money is too much and tell other women not to go. The reason are the nurses are not always on duty for their primary assignment [CC07, Esan South East].*

Participants cited nurses’ private practices as a reason for high absenteeism. They explained that health care staff would often operate their private clinics when they were scheduled to work at the community’s health facility. They would also refer patients from the community health clinics to their private clinics.

*May God keep you all, the health centre that they said is here there is no nurse where three nurses are supposed to be on duty is only one nurse you will see, in a week you will not see them if someone sustain any injuring and be rush there you will not see nurses except you will go to the next community [CC08, Esan South East].*

Participants expressed the need for community health facilities to be constantly monitored by the government. They felt that the health care staff’s attitude to their work was as a result of poor monitoring and accountability by the government. Quality of care notwithstanding, participants acknowledged the importance of seeking skilled health care during pregnancy and childbirth. They expressed confidence in the hospital’s ability to handle pregnancy-related complications such as preterm delivery. One participant believed that women’s utilization of skilled care facilities would prevent maternal deaths in the community.

*… enlighten them to go to the hospital for antenatal during pregnancy period. If we can take this step, this issue of death during childbirth will be abolished my major contribution here is educating the woman which is one most important tool. If we have health centre now, is to tell the women to go to the health centre so that at the end, the woman will deliver safely so that’s my contribution. I am a man with children, in cases where there is miscarriage, the husband and the wife are not happy about it so at the end, when the maternal death is no more among our women, we will give God the glory for that [CC01, Esan South East].*

Some participants commented on poor quality care in their community’s health facility. They opined that it was not enough to have a health care facility, the nurses also need to be knowledgeable in order to assist women. One participant called for nurses to be retrained.

*{There were 15 items that were donated to that hospital, but I can tell you that all these things. I mentioned, the so-called nurses were seeing them for the first time. I don’t think they have ever used that equipment since it was brought there, there was another machine there, that is supposed to be use for checking sugar level, the nurses there I don’t think they know how to use it, so what I am saying in essence is that these nurses themselves who are supposed to be the ones helping us, they need to be trained, they need to be up to date with the recent equipment you have in the world today, they need to update themselves and I know that is the functional utility of a government to intermittently bring your working personnel up to date with the machine you give to them to work, so training number 1}* [CC01, Etsako East ]

### Distance to health facilities

Some participants reported not having primary health care (PHC) facilities in their communities. They would often need to travel long distances to a neighboring community to access the nearest PHC.

*This is my own and this is our own there is a big difference if we have our own maternity here we will not be going outside to look for another care elsewhere. If we have health centre here whenever a woman is under labour no need of going too far, she is just to be taken to our health here and deliver freely and healthy with no bad news or stress rather than joy of a mother. Please this is what we are pleading for please come to our aid is long this community has been suffering (CC02 Etsako East).*

Inaccessible roads and lack of transportation also made it difficult to reach the nearest health facility during an emergency. Lack of access to health facilities was a major challenge and participants stated that these issues have resulted in loss of lives within the community.

*We that you are seeing here now, if we need ordinary bike (motor cycle) to come out from here, its difficult for us. Sometimes, if our wives fall into labour at night, before we can come out from here to access the health centre at Eguare (next PHC community), it will be very difficult. You now see that the actual time a woman would have delivered will now be prolonged because she does not arrive early. Sometimes, women give birth on their way. Even, children, like the other day that a child was wounded, to take him to general hospital, before we could get motor cycle, it was too late, we got to general hospital late at night before they could treat him. So many things. That’s why you see small sickness will be killing children. These are the major problems that we have here. Thank you [CC03, Esan South East].*

In general, the participants demonstrated awareness about the benefits of skilled maternal healthcare services, particularly for pregnancy and childbirth. They welcomed initiatives to that end and pledged to support such initiatives.

*I have told you four villages live together here. If there is health centre here, it’s going to be very busy because women from all the communities will be using it. If there is health centre here we will be very happy that good thing has come to our land and we are ready to corporate with you in any way to see that everything goes fine. Even if you want to start it today, we have a vacant building, many vacant buildings here…where even if you turn it to maternity, no payment is needed for the building, you go ahead and use it. That is my contribution [CC03, Esan South East].*

*Like the last one you are saying now, Insurance and co-sharing. We like it. We have been having community money; we can use it to help in urgent needs of pregnant women and children. Like you come now, if women have been here, they would have been able to speak for themselves. You know women have a way of having their own caucus. If women want to deliver, or want to get pregnant, these are the things they need to talk about [CC04, Esan South East].*

## Discussion

This study fills a gap by providing rich descriptions of male elders’ perceived barriers to maternal healthcare access and usage thereby augmenting the existing literature on male involvement in MNCH in Nigeria. These barriers also hindered male participation in MNCH. In patriarchal societies, men play an important role in facilitating timely maternal access to appropriate health care. Their involvement in various aspects of maternal health including contraceptive use, support for women during pregnancy, the use of evidence-based care for antenatal, delivery and postnatal care, can address gender influences on maternal health outcomes. It is therefore important that men’s sphere of influence is understood so that opportunities to improve women’s health are negotiated [30].

### Community barriers

Findings from this study indicate that male involvement was rooted in gender roles where men continue to be viewed as financial providers and decision makers. Men reported having no interest in being present during childbirth. Furthermore, information on family planning was considered “women’s responsibility” and knowledge on family planning among the men was generally low. While some participants recounted instances where they accompanied their wives to health facilities for delivery, responses indicate a dichotomy in gendered expectations of men and women. A husband determined the method of care his spouse received, which in turn depended on his financial status. This reinforces the gendered view of men as providers and decision makers. Participants opined that women whose husbands were poor were more likely to patronize traditional birth attendants because they were cheaper compared to health facilities. A similar study reported an association between a woman’s use of maternal services and her husband’s income [52]. It was also suggested that having social and community interactions can increase men’s awareness on maternal health issues and modify their behavior.

Majority of the men were willing to enable their wives’ access to skilled maternal healthcare. Men were more likely to express financial support and were less likely to express support in birth preparedness and postnatal care. Similar studies have indicated men’s exclusive interest in financial matters related to maternal health care [53]. Strategies to actively engage men in all spheres of maternal health deliberately target patriarchal structures that reinforce pre-existing norms. These strategies have involved behavior change, which is a sensitive topic particularly when involving outsiders and non-members of their community. A strategy in Malawi encouraged men to get into peer groups and participate in ANC educative classes. These classes encouraged men to accompany their wives to antenatal care visits [54].

Women’s use of traditional centres highlighted men’s role in women’s reproductive health. Findings from this study showed that women’s use of traditional birth attendants (TBA) for pregnancy care and delivery was influenced by men’s perception of TBA. There were mixed perceptions about women’s use of traditional centres. Some men described traditional healers as lacking in knowledge and equipment to manage complications during childbirth. They reported that their wives utilized skilled MNCH services for pregnancy care and delivery.

However, men who had positive perceptions of TBAs supported their wives’ use of TBA. For some, socio economic status played a role in their wives’ patronage of traditional healers; others held the skill and experience of TBAs in high regard. Elsewhere, researchers reported that when women lacked decision-making autonomy, their husbands determined their health seeking behaviors [55, 56]. Other factors such as distance to facilities and the lack of transportation, influenced women’s use of traditional healers for pregnancy care. Studies on how to increase maternal use of skilled care point out that several factors such as distance to health facilities, availability of transport and family choices need to be addressed [56, 57].

### Health systems

Men were generally aware of the benefits of skilled care and an overwhelming majority of them identified health systems failures as barriers to equitable access to care. Men’s responses indicated that long travel distances and lack of transportation to health facilities discouraged access to and utilization of health facilities. Poor service quality such as habitual absenteeism of nurses and poorly equipped facilities posed a major barrier to service use. Issues highlighted in this study are consistent with a report on the state of primary healthcare (PHC) facilities in Nigeria. The Centre for Population and Environmental Development (CPED) examined the state of PHCs across Nigeria [7]. The poor performance of PHCs particularly in rural areas was emphasized. The CPED explained that PHCs are provided by the local government authority and have been found to be largely nurse-based. PHCs are usually managed solely by a nurse who is occasionally assisted by a clerical staff. This can have implications on workload and quality of care provided. Consistent with our findings, the CPED revealed that primary healthcare settings in rural areas are disproportionately lacking in both equipment and human resources. Maternal deaths have been shown to decrease significantly when women were treated by diverse healthcare personnel [58].

Furthermore, while distance to facilities were identified as barriers, some men were willing to travel long distances with their wives to access quality care. Some, however, were not willing to travel the distance thereby causing their wives to seek other sources of healthcare. The cost of care was identified as a barrier to accessing maternal care. Interestingly, in as much as some of the men complained about the cost of MNCH services, they were willing to pay for quality care. This is one of the central arguments made by Thaddeus and Main in their “three delay model” [59]. Instances in Ghana and some parts of Nigeria have shown that despite the provision of free maternal care services, maternal usage of health services appears to have stagnated [60, 61]. This indicates the need to address the multiple barriers to maternal access and usage of health care services.

Community leaders play a crucial role in influencing the use of health services in rural communities. As evidenced from this study, the elders demonstrated the importance of maternal access and usage of skilled healthcare by suggesting various strategies. Education was one of their strategies to increase the uptake of MNCH services. Others proposed the use of incentives to encourage preventive behaviors during pregnancy and overall to encourage the use of MNCH services. There is evidence of strategies involving a reward system whereby community chiefs partnered with health centres to encourage men’s involvement in maternal health. Community leaders instituted bylaws whereby men were fined for not accompanying their wives to prenatal care visits [62]. Similarly, in rural Malawi, a collaboration between community leaders promoted male involvement in maternal health: couples who attended prenatal services together received priority service as a reward. Countries like Tanzania, Malawi and Zimbabwe where community elders implemented such fines have deemed it effective in improving MNCH service utilization [63].

While this study fills a gap by providing rich descriptions of men’s perceived barriers to maternal healthcare access and usage, it is not without its limitations. This study relied on self-reported behavior of men and not direct observations. However, the authors note that a variety of responses were reported in the findings.

## Conclusion

Despite evidence that male involvement increased uptake of maternal and child services among women, studies show that few men are participating in MNCH programs. Community leaders play a crucial role in influencing the use of health services in rural communities. Findings suggest that community elders are not only in a good position to influence men’s behavior; they are also a source of information to policy makers on strategies to overcome barriers to maternal health, especially at the community level. Furthermore, community elders need support to enact regulations that will promote men’s involvement in maternal health, thereby increasing maternal use of MNCH services.

## Data Availability

The datasets generated and/or analyzed during the current study are not publicly available due analysis being underway for subsequent publications. They are available from the corresponding author on reasonable request.

## Abbreviations

CC: Community conservations
CPED: Centre for Population and Environmental Development
LGAs: Local government areas
MNCH: Maternal, newborn, and child health
MSS: Midwives Service Scheme
PHCs: Primary health care services
SURE-P: Subsidy Reinvestment and Empowerment Programme
TBAs: Traditional birth attendants
